# Interleukin-19 Levels Are Increased in Palmoplantar Pustulosis and Reduced following Apremilast Treatment

**DOI:** 10.3390/ijms24021276

**Published:** 2023-01-09

**Authors:** Kerstin Wolk, Dagmar Wilsmann-Theis, Katrin Witte, Theresa-Charlotte Brembach, Christian Kromer, Sascha Gerdes, Kamran Ghoreschi, Kristian Reich, Rotraut Mössner, Robert Sabat

**Affiliations:** 1 Psoriasis Research and Treatment Center, Charité—Universitätsmedizin Berlin, 10117 Berlin, Germany; 2 Interdisciplinary Group of Molecular Immunopathology, Dermatology/Medical Immunology, Charité—Universitätsmedizin Berlin, 10117 Berlin, Germany; 3 Department of Dermatology and Allergy, University Medical Center Bonn, 53127 Bonn, Germany; 4 Department of Dermatology, Georg-August-University Goettingen, 37073 Goettingen, Germany; 5 Center for Inflammatory Skin Diseases, Department of Dermatology, University Medical Center Schleswig-Holstein Campus Kiel, 24105 Kiel, Germany; 6 Department of Dermatology, Venereology and Allergology, Charité—Universitätsmedizin Berlin, 10117 Berlin, Germany; 7 Center for Translational Research in Inflammatory Skin Diseases, Institute for Health Services Research in Dermatology and Nursing, University Medical Center Hamburg-Eppendorf, 20251 Hamburg, Germany

**Keywords:** defensin, IL-1, IL-17, chemokine, phosphodiesterase 4, small molecule

## Abstract

Palmoplantar pustulosis (PPP) is a chronic inflammatory skin disease characterised by neutrophilic granulocyte (neutrophil)-filled pustules on the palms and soles. The pathogenesis of PPP is poorly understood. This study conducted an identification of the immune mediators associated with PPP and an exploration of apremilast treatment effects on them. We screened for immune mediators elevated in blood taken from 68 patients with PPP versus control participants and included the most promising parameters in the protocol of phase the 2, multicentre study of apremilast (PDE4 inhibitor) in 21 patients with moderate-to-severe PPP (APLANTUS; EudraCT 2016-005122-11) for respective analysis of blood and skin samples of study patients. We investigated stimulated neutrophils and three-dimensional reconstituted epidermis cultures. Interleukin (IL)-19 was found to be the most upregulated immune mediator in the blood of PPP patients. IL-19 serum levels were independent of patients’ age, gender, and BMI but were associated with strongly upregulated cutaneous IL-19 expression and correlated with the number of palmoplantar pustules. In patients participating in the APLANTUS study, apremilast reduced pustules more effectively than erythema and scaling. Moreover, this treatment significantly reduced IL-19 blood and skin levels. The reduction in IL-19 blood levels at week 4 correlated with the reduction in pustule counts at week 20 (end of treatment). IL-19 was expressed by neutrophils activated in vitro and induced CXCL6, a neutrophil-attracting chemokine, in epidermis models. This work demonstrates elevated IL-19 levels in the blood and skin of PPP patients and suggests a relevant role of this cytokine in the appearance of pustules in this disorder. It also suggests the suitability of IL-19 blood levels as a predictive biomarker for the treatment response of PPP patients, which should be validated in further studies.

## 1. Introduction

Palmoplantar pustulosis (PPP) is a chronic inflammatory skin disease characterised by the recurrent appearance of pustules on the palms and soles located within erythematous and desquamating areas [1]. Relevant prevalence data come from Japan and Sweden and are in the range of 0.1–0.2% [2,3]. Palmoplantar involvement imposes major disabilities and is associated with pain and profound impairment of the quality of life [4]. A relevant proportion of PPP patients additionally suffers from hyper-cholesterolaemia (~38%), hypertension (~38%), thyroid disease (~28%), metabolic syndrome (~28%), psoriasis vulgaris (PsV; ~25%), and/or psoriatic arthritis (~22%) [4,5,6].

The pathogenesis of PPP is largely unclarified. Although PPP used to be assigned to psoriasis, evidence from both basic and clinical research indicates that the disease mechanisms in PPP differ from the well-understood mechanisms underlying PsV [7,8]. For example, PPP is not associated with *PSORS1*, the main susceptibility locus for PsV that contains the alleles for, e.g., HLA-Cw*6 [9]. As inferred from clinical studies, interleukin(IL)-17 or IL-23, two key cytokines of PsV [7], seem to play only a moderate role in PPP pathogenesis [10,11,12]. It was even recently reported that the T-cell response in skin lesions of PPP patients strongly overlaps with the T-helper (Th)2 cell response rather than with the Th17 or Th1 response [13]. PPP also differs from generalised pustular psoriasis since *IL36RN* mutations formative in this psoriasis form are very rare in PPP [14]. Instead, other innate immune mechanisms seem to play an important role in PPP, particularly those related to the infiltration and action of neutrophilic granulocytes (neutrophils) in the skin [15]. In these processes, the microbiome components recently found inside the “sterile” pustules of PPP patients [16] may be relevant.

Because of the limited understanding of the disease pathogenesis, innovative therapies and evidence-based guidelines for the management of PPP are lacking. This study therefore aimed at the identification and characterisation of immunological players in PPP.

## 2. Results and Discussion

**Figure 1:**
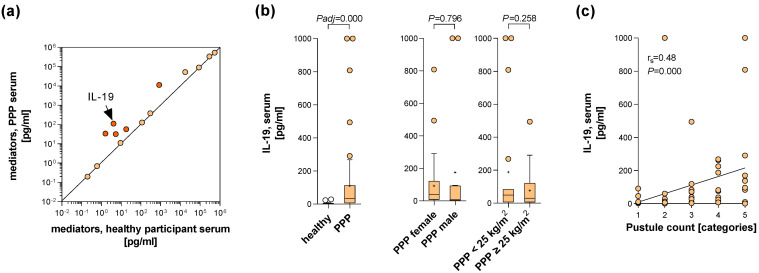
IL-19 serum levels are significantly upregulated in PPP patients. (**a**–**c**) Blood was collected from 68 PPP patients and 19 age- and sex-matched healthy control participants. Levels of 14 immune mediators were quantified in serum or plasma by ELISA (for the concrete number of samples analysed for each mediator, see Figure S1). (**a**) Mean levels of the 14 immune mediators quantified in serum or plasma from PPP patients and healthy participants were plotted against each other. (**b**) Serum IL-19 data from PPP patients and healthy participants are shown in Tukey-style box plots, with box centerline and “+” representing the median and mean, respectively. Differences between groups were analysed using the Mann-Whitney U-test (*p*-value adjusted using the Bonferroni method for multiple testing (screening of 14 serum parameters) indicated) (*left*). Serum IL-19 data from the PPP patients were divided based on sex (*middle*; 47 females, 13 males) and body mass index (BMI) (*right*; 20 normal-weight patients with BMI < 25 kg/m^2^, 40 pre-obese or obese patients with BMI ≥ 25 kg/m^2^) of patients. Data are shown in Tukey-style box plots. Differences between groups were analysed using Mann-Whitney U-test (*p*-values indicated). (**c**) Serum IL-19 data from 60 PPP patients were plotted against the pustule count category (pustule count on the most affected location categorised into the levels 1 to 5 corresponding to 0, 1–10, 11–25, 25–60, and > 60 pustules) of these patients and correlation between both parameters was tested by Spearman’s correlation test (correlation coefficients r_s_ and *p*-value indicated).

In order to obtain clues to key players in PPP, we screened for soluble mediators elevated in the blood that play a role in intercellular communication. Blood collected from 68 PPP patients (PPP cohort 1) and 19 healthy control participants was used for individual quantification of 14 mediators by means of enzyme-linked immunosorbent assay (ELISA). The parameter whose concentration was most increased in serum/plasma of PPP patients was IL-19 (Figure 1a and Figure S1; ~27-fold increase). This increase remained strongly significant after *p*-value adjustment for multiple testing (Figure 1b and Figure S1). IL-19 is a member of the IL-10 family of cytokines, which comprises mediators such as IL-20 and IL-22 with a key role in epidermis biology [17]. The analysis of the possible relationship of IL-19 serum levels to patient characteristics showed no association with age, gender, or body mass index of PPP patients (Figure 1b). Importantly, patients’ IL-19 serum levels correlated with the palmoplantar pustule counts (Figure 1c). These data suggested a role of IL-19 in PPP pathogenesis and the potential suitability of IL-19 as a biomarker for PPP disease severity.

**Figure 2:**
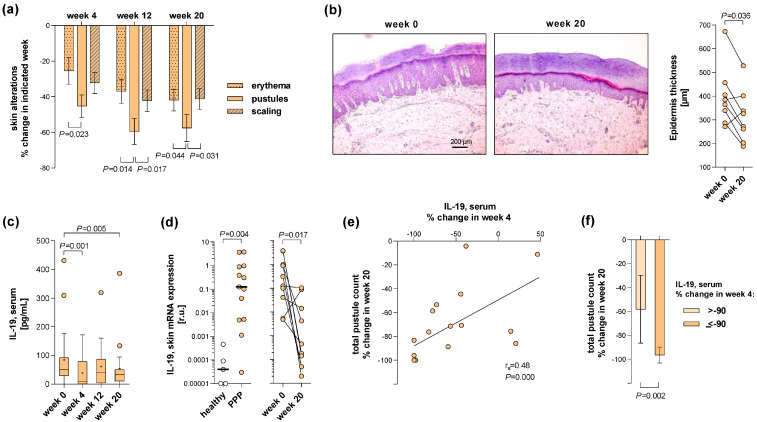
Early apremilast therapy-induced decrease in IL-19 serum levels may predict clinical response in PPP patients. In the scope of the APLANTUS study, patients with moderate to severe PPP were treated with apremilast for 20 weeks [18]. Disease severity was assessed using the PPPASI, a composite score based on the degree of specific skin alterations (pustules, erythema, and scaling) as well as lesion area and location [18]. Blood and (optionally) lesional skin samples were collected before (week 0) and at different time points after therapy started (week 4, week 12 (only blood), and week 20). (**a**) Percentage changes in individual skin alterations accounting for the PPPASI [18] (degree of pustules, erythema, scaling) observed in 20 patients at week 4, week 12, and week 20 relative to week 0 are indicated. Differences between changes in the individual alterations were analysed for each time point using the Wilcoxon matched-pairs signed-rank test (*p*-value indicated). (**b**) Paired lesional skin samples obtained from 8 patients before and at the end of treatment were analysed using paraffin-based histology. The thickness of living epidermal strata (down to the tips of rete ridges) was measured in haematoxylin and eosin-stained skin sections. Representative sections from one patient (*left*) and paired individual thickness data of the 8 patients (*right*) are presented. The difference between time points was analysed using Wilcoxon matched-pairs signed-rank test (*p*-value indicated). (**c**) Blood samples collected at different time points from 19 patients were analysed for serum IL-19 using ELISA. Data are presented as Turkey-style box plots, with box centerline and “+” representing the median and mean, respectively. Changes during therapy were analysed using the Wilcoxon matched-pairs signed-rank test (*p*-values indicated). (**d**) Skin samples were subjected to IL-19 mRNA expression analysis using RT-qPCR. *Left:* Results obtained with skin samples taken from lesions of 13 PPP patients and palmar areas from 5 healthy control participants are presented as individual and median (black bars) data (relative units, r.u.). The difference between groups was analysed using the Mann-Whitney U-test (*p*-value indicated). *Right*: Results obtained with paired lesional skin samples obtained from 9 PPP patients before and at the end of treatment are presented as individual data (relative units, r.u.). The difference between time points was analysed using the Wilcoxon matched-pairs signed-rank test (*p*-value indicated). (**e**) Percentage change in patients’ serum IL-19 levels at week 4 relative to week 0 (n = 17) and percentage change in patients’ total pustule counts at week 20 relative to week 0 were calculated and plotted against each other. The correlation between both parameters was tested by Spearman’s correlation test (correlation coefficients r_s_ and *p*-value indicated). (**f**) Patients were divided into two groups depending on the achieved percentage change in IL-19 serum levels at week 4 of treatment (see (**e**)), the one with a percentage change of > −90% (e.g., reduction by less than 90%) and the other with percentage change of ≤ −90% (e.g., reduction by 90% and more). The difference between both groups was analysed using the Mann-Whitney U-test (*p*-values indicated).

Based on these data, we were interested in the behaviour of IL-19 in PPP patients during therapy. Thus, quantification of IL-19 serum levels was included in the protocol of the APLANTUS study as one out of eight serum/plasma parameters. APLANTUS is an open-label, phase 2, single-arm, multicentre study in which 21 patients with moderate-to-severe PPP (PPP cohort 2) were treated with apremilast for 20 weeks [18]. Apremilast is a phosphodiesterase-4 inhibitor and has been suggested to modulate the production of multiple immune mediators [19]. As APLANUS showed, apremilast treatment led to a significant decrease in total pustule counts per patient, with a median reduction of 76.3% at week 20 compared to baseline (*p* < 0.001) [18]. Furthermore, the Palmoplantar Pustulosis Area and Severity Index (PPPASI) decreased significantly, with a median reduction of 57.1% at week 20 (*p* < 0.001) [18]. Interestingly, analysing the changes in individual skin alterations accounting for PPPASI [18], we found that apremilast reduced pustules more effectively than erythema and scaling (Figure 2a). The study protocol included an optional donation of skin samples at week 0 (baseline) and week 20. Apremilast treatment also reduced epidermal acanthosis at week 20 compared to week 0 in 6 out of 8 patients [for *n* = 8: mean ± SD: 314.4 ± 111.4 µm (week 20) versus 397.6 ± 126.7µm (week 0), *p* < 0.036] (Figure 2b). Moreover, a drop in the cutaneous T-cell count was observed in 5 out of 8 patients (Figure S2). The analysis of blood samples collected before and at different time points of apremilast treatment demonstrated significantly reduced IL-19 levels at week 4 and at week 20 compared to week 0 (Figure 2c).

We then investigated whether IL-19 was produced in the inflamed skin of these patients and, if so, whether its production could also be inhibited by the Apremilast treatment. The analysis of skin samples collected at week 0 from lesions of the PPP patients and, for comparison, from the palms of matched healthy control participants, using RT-qPCR analysis, demonstrated strongly upregulated IL-19 levels in PPP lesions (Figure 2d). Furthermore, RT-qPCR analysis of paired biopsies collected from lesional PPP skin revealed a significant reduction of IL-19 mRNA levels at week 20 compared to week 0 (Figure 2d).

The next evaluation step addressed the question of whether the reduction of serum IL-19 levels was already detectable before the clinical improvement of the patients. Correlation analyses revealed a significant association between the percentage change in IL-19 serum levels at week 4 of treatment and the percentage change in total pustule count at week 20 (Figure 2e). Moreover, when the patients were divided into two groups depending on the achieved change in IL-19 serum levels at week 4, the one with a percentage change of>−90% (e.g., reduction by less than 90%) and the other with a percentage change of ≤−90% (e.g., reduction by 90% and more), a significant difference in the percentage change in pustule count at week 20 between the two groups was found (Figure 2f). This suggested that IL-19 serum levels in PPP patients might also be suited as a predictive biomarker for the treatment response of these patients; patients showing a clear IL-19 serum level reduction within the first weeks of apremilast treatment might benefit more from that therapy.

**Figure 3:**
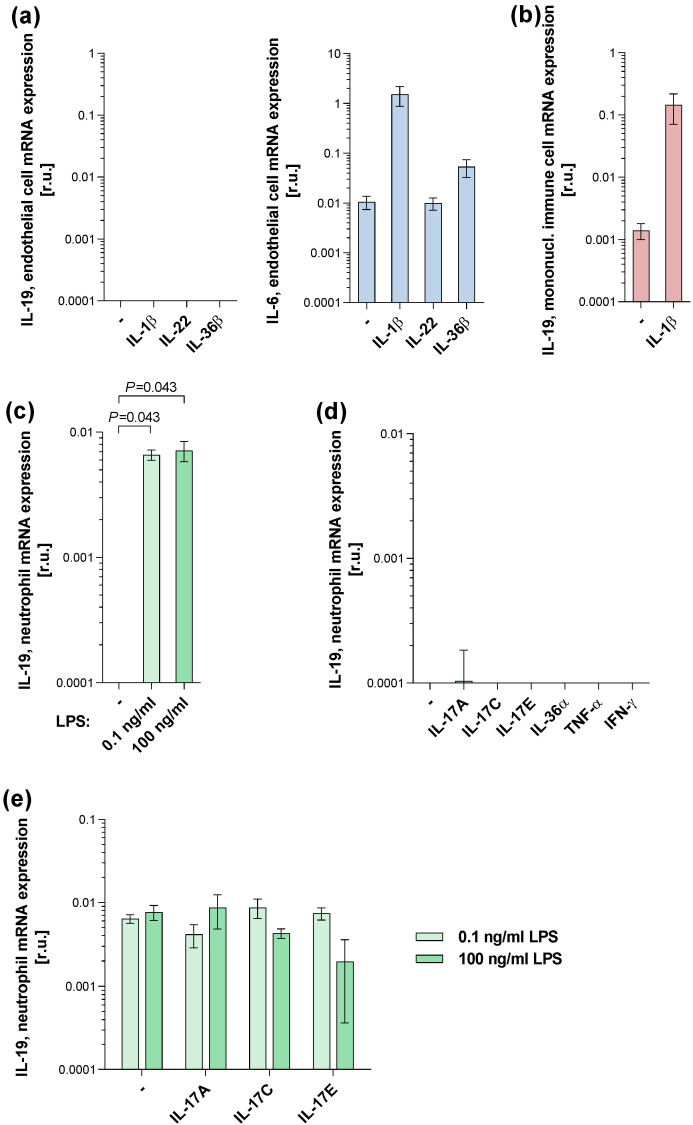
IL-19 is produced by neutrophils stimulated by microbial products. (**a**) Endothelial cells isolated from the skin of healthy donors were stimulated or not by IL-1β, IL-22, and IL-36β for 24 h. Cellular expression of IL-19 and IL-6 was quantified by RT-qPCR. Mean ±SEM data (relative units, r.u.) of 3 experiments are presented. (**b**) Mononuclear immune cells isolated from the peripheral blood of healthy donors were stimulated or not by IL-1β for 24 h. Cellular expression of IL-19 was quantified by RT-qPCR. Mean ±SEM data (relative units, r.u.) of 4 experiments are presented. (**c**,**d**) Neutrophils isolated from the blood of healthy donors were cultured with different concentrations of bacterial lipopolysaccharides (LPS) for 4 h ((**c**); 5 experiments) or in the presence and absence of IL-17A, IL-17C, IL-17E, IL-36α, TNF-α, or IFN-γ for 24 h ((**d**); 2–4 experiments). Cellular IL-19 mRNA expression was quantified using RT-qPCR. Mean (± SEM) data (relative units, r.u.) are presented. The difference between the untreated and LPS-treated groups was analysed using the Wilcoxon matched-pairs signedö-rank test (*p*-value indicated). (**e**) Neutrophils isolated from the peripheral blood of healthy donors were stimulated or not by IL-17A, IL-17C, and IL-17E for 24 h. For the last 4 h of the cytokine stimulation period, bacterial lipopolysaccharides (LPS, at indicated concentrations) were added to the cultures. Cellular expression of IL-19 was quantified by RT-qPCR. Mean ± SEM data (relative units, r.u.) of 2 to 4 experiments are presented.

We then returned to the bench to shed light on the potential cellular sources and action of IL-19 in PPP lesions. We have previously shown that, among immune cells, IL-19 is produced by activated monocytes [20] and, to a lower extent, activated macrophages and dendritic cells [21], but not by T or NK cells [20]. Furthermore, we have discovered that keratinocytes, after stimulation with IL-1β and IL-17A, as well as, to a lesser extent, tumour necrosis factor (TNF)-α, IL-4, and IL-22, express IL-19 [22,23], express IL-19. Now, we analysed dermal endothelial cells stimulated with different cytokines. While IL-19 expression was detected in mononuclear immune cells used as controls, no IL-19 expression was found in endothelial cells (Figure 3a,b). Stimulated by the observed link between IL-19 levels and pustule counts in PPP patients, we then investigated whether neutrophils were able to express IL-19. Indeed, a strong IL-19 expression was found after stimulation of these cells with even small amounts of microbial products (Figure 3c) but not with inflammatory cytokines like IL-17A, IL-17C, IL-17E, IL-36α, TNF-α, or IFN-γ (Figure 3d). IL-17A, IL-17C, and IL-17E did not strengthen the IL-19 expression induced by microbial products (Figure 3e).

**Figure 4:**
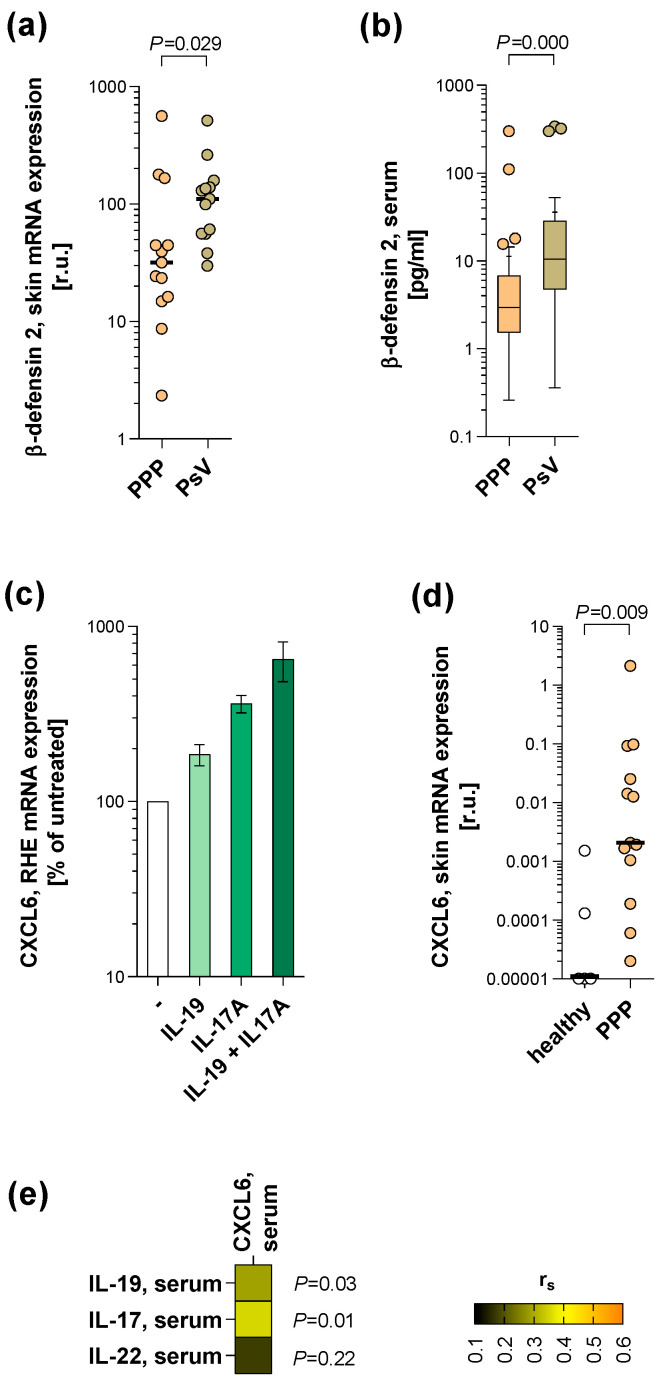
IL-19 may contribute to skin infiltration by neutrophils. (**a**) Beta-defensin 2 mRNA expression was quantified in samples collected from the lesional skin of 13 PPP patients and 13 PsV using RT-qPCR. Individual and median (black bars) data (relative units, r.u.) are presented. The difference between groups was analysed using the Mann-Whitney U-test (*p*-value indicated). (**b**) Serum β-defensin 2 levels were analysed in samples collected from 60 PPP patients and 44 PsV patients. Data are shown in Tukey-style box plots, with box centerline and “+” representing the median and mean, respectively. Differences between groups were analysed using the Mann-Whitney U-test (*p*-value indicated). (**c**) Reconstituted human epidermis samples (RHE) were cultured with IL-19, IL-17A, the combination thereof, or without stimulation for 72 h. The cellular mRNA expression of CXCL6 was quantified with RT-qPCR. Mean (±SEM) data of 3 experiments, relative to the non-stimulated group, are presented. (**d**) CXCL6 mRNA expression was quantified in samples collected from the lesional skin of 13 PPP patients and palmar skin from 5 healthy control participants using RT-qPCR. Individual and median (black bars) data (relative units, r.u.) are presented. The difference between groups was analysed using the Mann-Whitney U-test (*p*-value indicated). (**e**) Blood serum levels of CXCL6, IL-19, and IL-17 of PPP patients were quantified by ELISAs. The correlation of CXCL6 versus IL-19, IL-17, and IL-22 levels was tested using Spearman’s correlation test (n = 52–54). Correlation coefficients r_s_ are presented as a heat map (*p*-values indicated).

Induction of neutrophil IL-19 production by microbial products may be relevant for PPP, given the observation of microbiome components found in the “sterile” pustules of patients [16]. Interestingly, in PsV, the invasion of microbes into the disturbed epidermis is prevented due to massive keratinocyte production of antimicrobial proteins in these patients [24,25]. We, therefore, analysed the levels of β-defensin 2, the major inducible antimicrobial protein in the skin [25], in skin lesions of PPP versus PsV patients. As shown in Figure 4a, β-defensin 2 mRNA expression in PPP was clearly below that in PsV. Accordingly, we found lower blood serum levels of β-defensin 2 in PPP patients (PPP cohort 1) compared to PsV patients (Figure 4b). Whether other antimicrobial proteins are also limited in PPP lesions should be investigated in further studies.

IL-19 acts on its target cells via a transmembrane receptor composed of the IL-20R1 and IL-20R2 receptor subunits [17]. Human immune cells do not express the IL-20R1 subunit and do not respond to the presence of IL-19 [21,22]. In contrast, both receptor subunits are expressed by keratinocytes [22,23]. Since IL-19 can strengthen IL-17A effects and keratinocytes stimulated by IL-17 are able to produce chemokines to support cutaneous immune cell infiltration [23], we asked whether IL-19 is also able to induce neutrophil-attracting chemokines in keratinocytes. Using a 3-dimensional reconstituted epidermis, IL-19 was found to increase the expression of CXCL6 and to strengthen the CXCL6-inducing IL-17A effect (Figure 4c). In line with these data, CXCL6 levels in skin lesions of PPP patients were greatly elevated compared to the levels in the skin of healthy control participants (Figure 4d). The relevance of the IL-19/IL-17-induced CXCL6 production in PPP was further supported by the correlation of CXCL6 levels with IL-19 and IL-17A levels, but not IL-22 levels, in the blood of PPP patients (PPP cohort 1) (Figure 4e).

These data suggest a positive feedback mechanism for PPP pathogenesis, such that activated neutrophils in the patients’ inflamed skin produce IL-19, which in turn increases the influx of further neutrophils from the blood into the skin. Evidence of the role of IL-19 in PPP has also been provided from genetic investigations. Using a small initial cohort of 43 PPP patients, Kingo et al. suggested that specific polymorphisms in the IL19 gene cluster may influence susceptibility to PPP [26].

A limitation of our study is that the screening of parameters elevated in the blood of PPP patients was limited to 14 parameters. On the other hand, corresponding measurements of these parameters were carried out individually with high-quality ELISAs. The main limitation of our work is the small number of patients in the APLANUS study [18]. The number of samples for laboratory analysis, especially skin analysis, was, therefore, relatively small. In addition, the design of the APLANUS was a non-randomised and open-label design [18].

In summary, this study provides a new insight into the PPP pathogenesis, demonstrating the role of IL-19 in the appearance of pustules in this disorder. Thus, IL-19 may be a promising therapy target candidate for PPP. Moreover, it suggests the suitability of IL-19 blood levels as a predictive biomarker for treatment response in PPP patients, particularly those with high IL-19 levels and a predominant pustular component. Both aspects should be validated in further studies.

## 3. Materials and Methods

### 3.1. Samples from PPP Patients and Control Participants

The study was conducted according to the guidelines of the Declaration of Helsinki on Ethical Principles for Medical Research.

For the screening of blood immune mediators (Figure 1a–c and Figure S1), blood was obtained from 68 PPP patients visiting dermatological departments of different German university hospitals and from 19 healthy control participants. For comparison of β-defensin 2 blood levels between PPP patients and PsV patients (Figure 4b), additional blood was obtained from 44 PsV patients. The demographic and clinical characteristics of these participants are presented in Table 1.

The APLANTUS trial (EudraCT 2016-005122-11) is a phase 2, single-arm, multicentre study of apremilast in 21 subjects with moderate-to-severe PPP. The trial protocol, characteristics of the patients, and disease severity assessment were described in detail by Wilsmann-Theis et al. [18]. The trial protocol included the collection of blood at week 0 (baseline), week 4, week 12, and week 20 (end of treatment), as well as an optional donation of skin samples at week 0 and week 20.

For comparison of skin analysis data (Figure 2d and Figure 4a,d), control skin biopsies were taken from the palmar area of 5 healthy control participants and from the lesional skin of 13 PsV patients. The demographic and clinical characteristics of these participants are presented in Table 2.

For investigations outside the APLANTUS trial, respective data collection, sampling and analyses were approved by the institutional review boards (Ethikkommissionen) of the Georg August University, Goettingen, Germany [for PPP patients (24/03/09)] and the Charité University Medicine, Berlin, Germany [for healthy control participants (EA1/184/08, EA2/254/18) and PsV patients (EA1/213/08, EA1/321/12)], and written informed consent was obtained from all participants.

For investigations as part of the APLANTUS trial, respective data collection, sampling, and analyses were performed according to the study protocol that was approved by the institutional review boards [leading board: Ethikkommission der Medizinischen Fakultät der Rheinischen Friedrich-Wilhelms-Universität Bonn (352/17-AMG-ff)]. Written informed consent was obtained from all study participants.

### 3.2. Cell Cultures

Peripheral blood mononuclear cells were freshly separated from the venous blood of healthy donors by density gradient centrifugation, as previously described [27], and were stimulated with 10 ng/mL IL-1β or were left without stimulation (control) for 24 h. Dermal microvascular endothelial cells were obtained from Provitro and Life Technologies (Darmstadt, Germany) and cultured in an endothelial cell proliferation medium (Provitro), as recommended by the manufacturer’s protocol. Cells were stimulated with 5 ng/mL IL-1β, 20 ng/mL IL-22, and 50 ng/mL IL-36β or were left without stimulation (control) for 24 h.

Human neutrophils were isolated from citrated venous blood of healthy donors using a procedure adapted from that described by Nauseef WM et al. [28]. In brief, blood was subjected to a density gradient centrifugation (40 min, 390× *g*) using conical centrifuge tubes and Ficoll–Paque Plus (1.077 g/mL; Biochrome). The formed erythrocyte/neutrophil layers were resuspended in phosphate-buffered saline (PBS) and mixed with an equal volume of 3% Dextran (Mr 450,000-650,000; Merck/Sigma-Aldrich) and let stand in an upright position at room temperature for 30 min to allow sedimentation of erythrocytes. The formed upper neutrophil-rich layer was recovered and washed in PBS (10 min, 210× *g*). The remaining erythrocytes were lysed using distilled water, and natural osmolality was adjusted using an equal volume of 1.8% NaCl solution. Obtained neutrophils were washed in PBS. Their purity was determined as previously described [29]. Culturing of neutrophils was done in supplemented VLE RPMI 1640 medium with/without 33 ng/mL IL-17A, 33 ng/mL IL-17C, 33 ng/mL IL-17E, 50 ng/mL IL-36α, 5 ng/mL tumour necrosis factor(TNF)-α, and 10 ng/mL interferon(IFN)-γ for 24 h, with/without 0.1 or 100 ng/mL LPS (*E. coli* 0111:B4; Sigma) added for the last 4 h of culturing.

To explore IL-19 effects on human keratinocytes, EpiDerm^TM^-201 underdeveloped reconstituted human epidermis (Mattek) was cultured in inserts at the air–liquid interface as described previously [30] and was stimulated or not (control) with 100 ng/mL IL-19, 10 ng/mL IL-17A, or combinations thereof for 72 h.

All recombinant cytokines used for cell stimulation studies described above were purchased from R&D Systems. After culturing, cells and epidermis models were lysed and frozen for RT-qPCR analysis.

### 3.3. Quantitative mRNA Expression Analysis

Cell lysis and skin tissue homogenisation, isolation of total cellular RNA, reverse transcription of mRNA, and quantitative gene expression analysis by TaqMan^TM^-based PCR (RT-qPCR) using exon–exon spanning fluorescent probes and the ABI Prism 7700 Sequence Detection System or StepOnePlus TM (both from Applied Biosystems) were carried out as described previously [20,31]. The assays for human IL-19, IL-6, CXCL6, and the housekeeping genes hypoxanthine phosphoribosyl-transferase 1 (HPRT1) and glyceraldehyde-3-phosphate dehydrogenase (GAPDH, used for neutrophil analyses) were purchased from Applied Biosystems. PCR was run in triplicates and in parallel with that of housekeeping genes. Based on identical amplification efficiencies, the detected threshold cycle values of target genes versus housekeeping genes were used to calculate normalised expression levels (relative units, r.u.) [32].

### 3.4. Histology and Immunohistochemistry

Sections of formalin-fixed and paraffin-embedded skin samples were sectioned on a microtome at 3 µm, deparaffinised in xylene or Roticlear (Roth), and rehydrated through a graded series of alcohols to deionised water before staining. For epidermal thickness determination, slides underwent staining by haematoxylin (Sigma) and eosin G (Roth). Image capture was conducted using an Axioplan imaging fluorescence microscope (Zeiss). Epidermal measurements (distance from the top of the living epidermis to the end of the rete ridge) were done using microscope-associated AxioVision software version 4.6.3. For CD3 staining, slides underwent heat-induced epitope retrieval in a citrated buffer (pH 6) at 110 °C for 20 min, followed by peroxidase block in Dako REAL peroxidase-blocking solution (DAKO) for 10 min. CD3 was detected using polyclonal rabbit anti-human CD3 antibody (DAKO; 1:100) and visualised using DAKO REAL Detection System (AP/RED, rabbit/mouse) (DAKO). Haematoxylin (Mayer’s haemalaun; Sigma) solution was used for tissue counterstaining. After image capture using a BZ-X800 Fluorescence microscope (Keyence), cell counting was performed using Fiji ImageJ software v2.9.0.

### 3.5. Enzyme-Linked Immunosorbent Assay

For the screening of PPP blood levels, the following immune mediators were individually quantified by enzyme-linked immunosorbent assays (ELISAs): lipocalin-2, β-defensin 2, IL-22, IL-20, IL-19, IL-1β, TNF-α, serum amyloid A (SAA), CCL2, CXCL6, resistin, chemerin, fetuin-A, and angiogenin. For SAA and β-defensin 2, ELISAs from Invitrogen and Immundiagnostik AG were used, respectively; all other assays were Quantikine™ ELISA systems obtained from R&D Systems (high sensitivity Quantikine™ variants for IL-1β and TNF-α). Quantification of β-defensin levels in serum obtained from PPP and PsV patients (as confirmation of in vitro results) was performed using Quantikine™ HS ELISA from R&D Systems. Blood samples from PPP patients participating in the APLANTUS study were subjected to Quantikine™ ELISAs from R&D Systems to individually quantify the following immune mediators: IL-19, IL-22, G-CSF, LCN2, MMP8, sP-selectin, SCF, sSCF-R.

### 3.6. Statistical Analysis

SPSS 19·0 software (IBM, Armonk, NY, USA) was used. The percentage change for a measured parameter value at week x of therapy was defined as (value at week x–value at week 0)/value week 0. Correlations were analysed using Spearman’s correlation test. The Mann–Whitney U-test (two-tailed) was used to analyse differences between PPP patients and healthy participants or PsV patients. The Wilcoxon matched-pairs signed-rank test (two-tailed) was used to analyse the differences between study time points in the APLANTUS study as well as the difference between the LPS-treated and untreated neutrophil granulocytes (minimum of five experiment repetitions).

## Figures and Tables

**Table 1:** Demographic and clinical characteristics of study participants who took part in the screening of blood immune mediators.

	PPP Patients (n = 68)	Healthy Donors (n = 19)	PsV Patients (n = 44)
Age (years), mean ± SD; range	49.5 ± 13.7; 18–78	43.8 ± 8.1; 33–58	39.0 ± 12.1; 19–64
Sex distribution (% female)	78	79	52
PPP pustule score *, mean ± SD; range	3.00 ± 1.23; 1–5	-	-
PASI	-	-	11.9 ± 5.7; 3.0–31.7
Disease duration (years), mean ± SD; range	7.6 ± 10.6; 0–43	-	15.9 ± 11.6; 1–42

* with pustules counted on the most affected location (e.g., left palm) and categorised into one of five levels (0, 1–10, 11–25, 25–60, >60 pustules).

**Table 2:** Demographic and clinical characteristics of study participants who donated control skin biopsies.

	Healthy Donors (n = 5)	PsV Patients (n = 13)
Age (years), mean ± SD; range	54.6 ± 8.8; 43–63	45.8 ± 12.4; 25–63
Sex distribution (% female)	80	15
PASI	-	15.7 ± 10.9; 6.1–36.5
Disease duration (years), mean ± SD; range	-	20.0 ± 11.6; 6–42

## Data Availability

The main data are presented within the figures of the manuscript. Further study material will be made available upon request by the corresponding authors to the extent permissible by law.

## Supplementary Materials

The following supporting information can be downloaded at: https://www.mdpi.com/article/10.3390/ijms24021276/s1.
