# Supplementary material for: Interleukin-19 Levels Are Increased in Palmoplantar Pustulosis and Reduced following Apremilast Treatment
Source: Int J Mol Sci. 2023 Jan 9;24(2):1276. doi: 10.3390/ijms24021276 (PMC9862858; doi:10.3390/ijms24021276)
Supplement: Supplementary file 1 [file ijms-24-01276-s001.zip › ijms-2104298-ori - SM.pdf]

International Journal of *Molecular Sciences*

MDPI

Article

Interleukin-19 Levels Are Increased in Palmoplantar Pustulosis and Reduced Following Apremilast Treatment

Kerstin Wolk [1,2,*,†], Dagmar Wilsmann-Theis [3,†], Katrin Witte [1], Theresa-Charlotte Brembach [1], Christian Kromer [4], Sascha Gerdes [5], Kamran Ghoreschi [6], Kristian Reich [7,‡], Rotraut Mössner [4,‡] and Robert Sabat [1,2,*,‡]

[1] Psoriasis Research and Treatment Center, Charité—Universitätsmedizin Berlin, 10117 Berlin, Germany
[2] Interdisciplinary Group of Molecular Immunopathology, Dermatology/Medical Immunology, Charité—Universitätsmedizin Berlin, 10117 Berlin, Germany
[3] Department of Dermatology and Allergy, University Medical Center Bonn, 53127 Bonn, Germany
[4] Department of Dermatology, Georg-August-University Goettingen, 37073 Goettingen, Germany
[5] Center for Inflammatory Skin Diseases, Department of Dermatology, University Medical Center Schleswig-Holstein Campus Kiel, 24105 Kiel, Germany
[6] Department of Dermatology, Venereology and Allergology, Charité—Universitätsmedizin Berlin, 10117 Berlin, Germany
[7] Center for Translational Research in Inflammatory Skin Diseases, Institute for Health Services Research in Dermatology and Nursing, University Medical Center Hamburg-Eppendorf, 20251 Hamburg, Germany
* Correspondence: kerstin.wolk@charite.de (K.W.); robert.sabat@charite.de (R.S.); Tel.: +49-03-450-518009 (K.W.); +49-03-450-518625 (R.S.); Fax: +49-03-450-518964 (K.W.); +49-03-450-518964 (R.S.)
† These authors contributed equally to this work.
‡ These authors contributed equally to this work.

Table S1. Screening for immune mediators with increased concentration in the blood of PPP patients. Blood was collected from 68 PPP patients and 19 age- and sex-matched healthy control participants. Levels of 14 immune mediators were quantified in serum or plasma by ELISA [PPP: n=60, except for IL-1β(n=29), TNF-α (n=26), CCL2 (n=51), angiogenin (n=56); control participants: n=19, except for TNF-α(n=13)]. Differences between PPP group and healthy control group were analyzed using Mann-Whitney U-test. For each mediator concentration, the fold increase observed for patient group compared to the healthy participant group and the P-value adjusted using the Bonferroni method for multiple testing are presented.

Immune mediator	Fold increase in PPP	*P*-value, unadjusted	*P*-value, adjusted
IL-1β	1.4	0.3428	1.0000
IL-19	26.9	0.0000	**0.0000**
IL-20	20.9	0.1038	1.0000
IL-22	6.0	0.0000	**0.0002**
TNF-α	1.2	0.4564	1.0000
CCL2	1.3	0.1239	1.0000
CXCL6	1.1	0.8815	1.0000
β-Defensin 2	13.3	0.0000	**0.0000**
Lipocalin 2	3.2	0.0000	**0.0000**
Angiogenin	1.1	0.1052	1.0000
Fetuin A	1.0	0.5432	1.0000
Chemerin	1.0	0.3960	1.0000
Resistin	1.2	0.0918	1.0000
Serum amyloid A	3.0	0.0016	**0.0221**

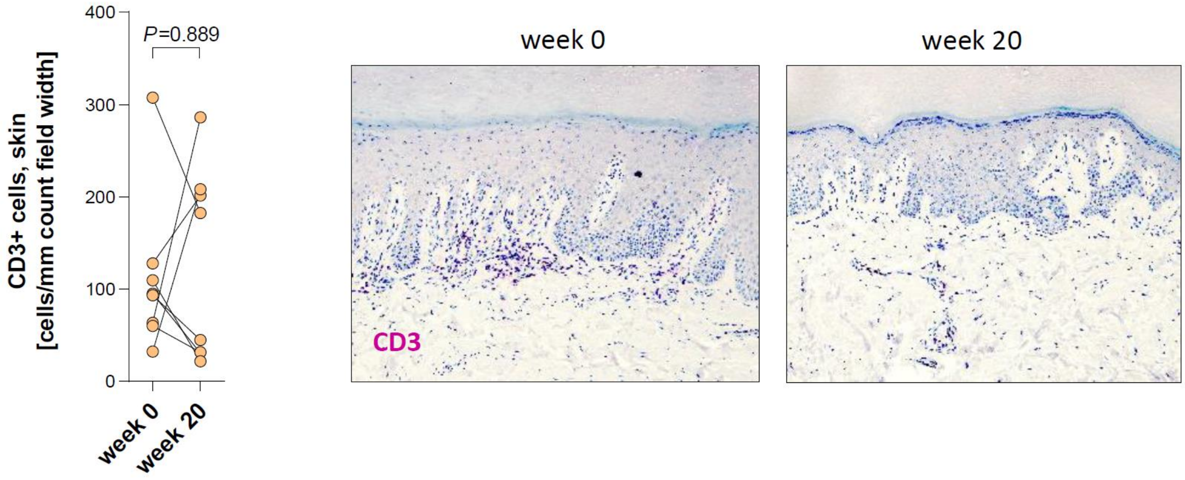

Figure S1. Cutaneous T-cell counts of apremilast-treated PPP patients. As part of the APLANTUS study, patients with moderate to severe PPP were treated with apremilast for 20 weeks as described in Wilsmann-Theis *et al.*, J Eur Acad Dermatol Venereol. 2021; 35(10):2045-2050. Paired lesional skin samples, obtained from 8 patients before (week 0) and at the end (week 20) of treatment, were analyzed using paraffin-based immunohistochemistry. T-cells were assessed by staining the skin sections with anti-CD3 antibody. Individual T-cell count data (*left*) and representative sections from one patient (*right*) are presented. The difference between time points was analyzed using Wilcoxon matched-pairs signed-rank test (two-tailed) (*P*-value indicated).
